# Modification of Pectin and Hemicellulose Polysaccharides in Relation to Aril Breakdown of Harvested Longan Fruit

**DOI:** 10.3390/ijms141223356

**Published:** 2013-11-27

**Authors:** Duoduo Wang, Haiyan Zhang, Fuwang Wu, Taotao Li, Yuxiang Liang, Xuewu Duan

**Affiliations:** 1 Key Lab of Plant Resources Conservation and Sustainable Utilization, South China Botanical Garden, Chinese Academy of Sciences, Guangzhou 510650, China; E-Mails: ddwang@scbg.ac.cn (D.W.); hy_zh07@163.com (H.Z.); 2004370322@163.com (F.W.); wubangcai88@163.com (T.L.); 2 Guangzhou No.6 Middle School, Guangzhou 510300, China; E-Mail: yxliang12@163.com

**Keywords:** aril breakdown, cell wall degradation, pectin, matrix glycan, depolymerization

## Abstract

To investigate the modification of cell wall polysaccharides in relation to aril breakdown in harvested longan fruit, three pectin fractions (WSP, water soluble pectin; CSP, CDTA-soluble pectin; ASP, alkali soluble pectin) and one hemicellulose fraction (4 M KOH-SHC, 4 M KOH-soluble hemicellulose) were extracted, and their contents, monosaccharide compositions and molecular weights were evaluated. As aril breakdown intensified, CSP content increased while ASP and 4 M KOH-SHC contents decreased, suggesting the solubilization and conversion of cell wall components. Furthermore, the molar percentage of arabinose (Ara), as the main component of the side-chains, decreased largely in CSP and ASP while that of rhamnose (Rha), as branch point for the attachment of neutral sugar side chains, increased during aril breakdown. Analysis of (Ara + Gal)/Rha ratio showed that the depolymerization of CSP and ASP happened predominantly in side-chains formed of Ara residues. For 4 M KOH-SHC, more backbones were depolymerized during aril breakdown. Moreover, it was found that the molecular weights of CSP, ASP and 4 M KOH-SHC polysaccharides tended to decrease as aril breakdown intensified. These results suggest that both enhanced depolymerization and structural modifications of polysaccharides in the CSP, ASP and 4 M KOH-SHC fractions might be responsible for aril breakdown of harvested longan fruit.

## 1. Introduction

Longan (*Dimocarpus longan* Lour.) is a typical non-climacteric subtropical fruit. It is one of the most important fruit crops in Southern China. However, the fruit quality deteriorates rapidly after harvest due to aril breakdown and decay development. Longan aril breakdown involves a loss of turgidity and translucency, resulting in tasteless fruit with reduced market values [1]. This physiological phenomenon is thought to resemble the softening of climacteric fruit, such as banana and papaya, which is the result of cell wall modification [2].

Plant cell wall is a complex matrix of polysaccharides, mainly consisting of pectin, hemicellulose and cellulose. Among these cell wall polysaccharides, hemicellulose is attached to cellulose microfibrils, forming the cellulose-matrix network that keeps the cell wall rigidity, while pectin matrix provides an environment for the deposition, slippage and extension of the cellulose-matrix network and is the major adhesive material between cells [3]. Depolymerization of pectin and hemicellulose plays an important role in fruit ripening, leading to the disassembly of cellulose and hemicellulose network and the decrease in fruit firmness. The involvement of polygalacturonase (PG), pectin methyl esterase (PME) and pectate lyase (PL) in the enzymatic disassembly of pectin polysaccharides have been extensively investigated [4]. Other enzymes that associate with hemicellulose disassembly include xyloglucan endotransglucosylase-hydrolase (XTH), endo-β-1,4-glucanase (EGase) and expansin (EXP) [5,6]. In addition, non-enzymatic degradation of cell wall polysaccharides may also account for fruit softening. Cheng *et al.* [7] and Duan *et al.* [2] suggest that the disassembly of cellular wall polysaccharides in banana and longan could be initiated by hydroxyl radical. Therefore, fruit softening is a result of cell wall modification caused by multiple factors.

The modification of cell wall polysaccharides caused by enzymatic and non-enzymatic factors includes the changes in composition, molecular weight and structural characteristics. There are some reports on the modification of cell wall polysaccharides in climacteric fruits such as apple [8], banana [9,10], papaya [11], peach [12], pear [13], plum [14], tomato [15] and melon [16]. However, variations in cellular wall compositions could lead to differences in softening-associated chemical modifications for each fruit species [17]. Further investigation into the degradation of pectin and hemicellulose with emphasis on structural modifications and characteristics in non-climacteric fruits during texture deterioration is required.

The objective of this study was to investigate the changes in monosaccharide compositions and molecular mass distributions of cell wall polysaccharides in longan fruit during storage, which will help understand aril breakdown of longan in the term of modification of cell wall polysaccharides.

## 2. Results and Discussion

### 2.1. Aril Breakdown and Cell Wall Material

**Figure 1.**
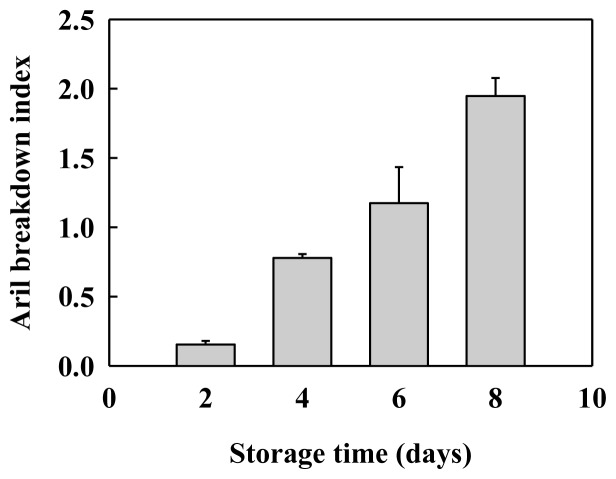
Change in aril breakdown index of longan fruit during storage at 25 °C. Data were presented as means ± standard errors (*n* = 3).

**Figure 2.**
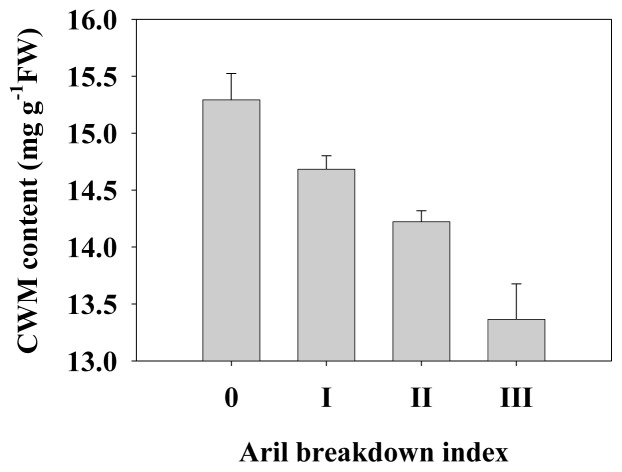
Cell wall material content in harvested longan fruit with different aril breakdown indices. Data were presented as means ± standard errors (*n* = 3).

Longan (*Dimocarpus longan* Lour.) is a typical non-climacteric fruit. Harvested longan fruit are characterized by rapid aril breakdown [1]. As shown in Figure 1, the aril breakdown index reached 1.95 after 8 days of storage at 25 °C and the fruit would lose commercial value. Furthermore, longan aril breakdown was accompanied by the decrease of cell wall material (Figure 2), indicating that cell wall degradation occurred in longan fruit during texture deterioration. In some climacteric fruits such as papaya [11], plum [18] and pear [13], fruit softening is associated with cell wall disassembly. Our results suggest that longan aril breakdown might be a consequence of the degradation of cell wall components.

### 2.2. Cell Wall Polysaccharides

**Figure 3.**
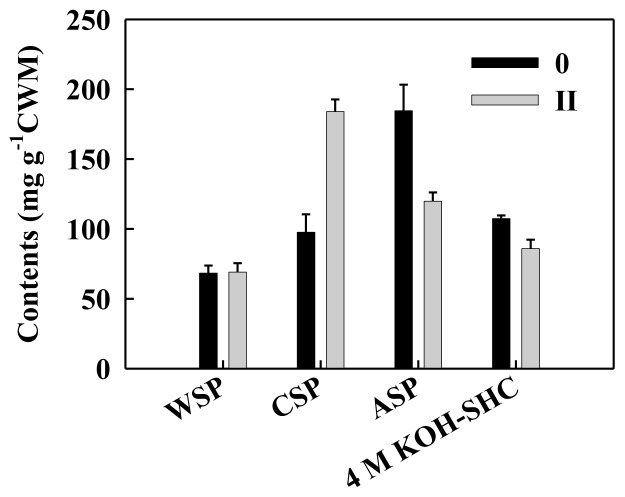
The contents of water soluble pectin (WSP), CDTA-soluble pectin (CSP), alkali soluble pectin (ASP) 4 M KOH-soluble hemicellulose (4 M KOH-SHC) extractedfrom aril tissue of longan fruit with different breakdown indices (0, no aril breakdown; II, moderate aril breakdown). Data were presented as means ± standard errors (*n* = 3).

Pectin is one of the major components in the primary cell wall and middle lamella. The degradation of pectin polysaccharides plays a key role in fruit softening [19]. As shown in Figure 3, ionically bound CSP content increased significantly, accompanied by a dramatic decrease of covalently bound ASP content from no aril breakdown to moderate aril breakdown, indicating that the solubilization and conversion of pectin polysaccharides might occur. However, loosely bound WSP content showed no differences between aril tissues with 0 and II grade breakdown. Some studies have shown that fruit softening is related to the increased WSP content [20,21]. In the present study, the decrease of ASP content was more likely to be important for the aril breakdown of longan fruit. Moreover, it is assumed that the increase of CSP mainly came from ASP during texture deterioration of longan fruit. Duan *et al.* [2] reported that application of exogenous hydroxyl radical accelerated aril breakdown and the conversion from ASP to CSP in longan fruit. Manrique and Lajolo also suggest that the solubilization of cellular wall polysaccharides is involved in the displacement of polymers from one fraction to another due to the modification of their structures [11].

For some fruit species, hemicellulosic matrix is as important as pectin matrix in determining the structural integrity of the cell wall [22]. In many cases, the disassembly of hemicellulose matrix is even more closely correlated with fruit softening [19]. In the present study, 4 M KOH-SHC content tended to decrease from no aril breakdown tissue to moderate aril breakdown tissue (Figure 3). Many previous studies have demonstrated the disassembly of hemicelluloses in ripening fruits such as apricot [21], banana [9] and peach [23].

Primary cell wall structure has been understood in general. It is thought that cellulose microfibrils embedded in varied matrix of pectin and glycan polysaccharides. There are reports of covalent linkages between xyloglucan and the arabinan/galactan side chains of rhamnogalacturonan-I [24,25]. In this study, the contents of covalently bound ASP and 4 M KOH-SHC tended to decrease as increased aril breakdown, implying that degradation of ASP and hemicellulose play a key role in aril breakdown of longan fruit. It is suggested that the breakdown of both ASP and hemicellulose disrupted cellulose-matrix network and reduced the integrity of cell wall and thereby led to cell collapse in longan fruit, and finally the tissue appeared autolysis.

### 2.3. Monosaccharide Compositions of Pectin and Hemicellulose Polysaccharides Fractions

**Figure 4.**
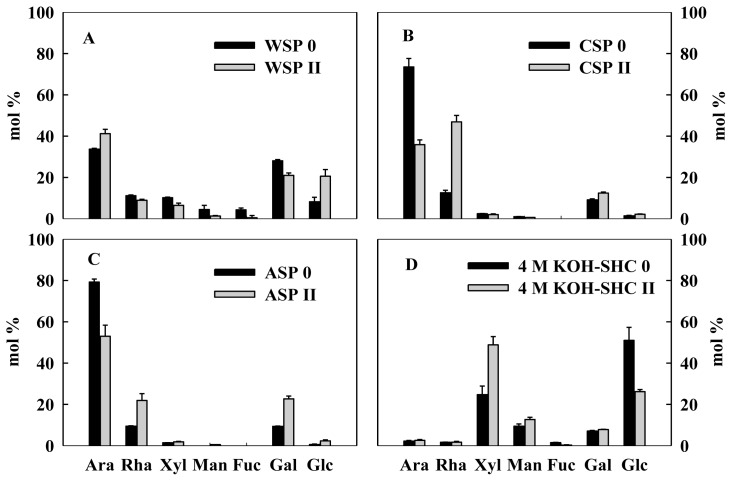
Neutral sugar compositions (mol%) of WSP (**A**), CSP (**B**), ASP (**C**) and 4 M KOH-SHC (**D**) polysaccharide fractions from aril tissues of 90 longan fruit with different breakdown indices (0, no aril breakdown; II moderate aril breakdown). Data were presented as means ± standard errors (*n* = 3).

The main neutral sugar compositions are indicative of the structure property of cell wall polysaccharides [11]. Figure 4 showed the molar percentages of arabinose (Ara), rhamnose (Rha), xylose (Xyl), galactose (Gal), glucose (Glc), mannose (Man), fucose (Fuc) from three pectin and one hemicellulose fractions in longan fruit. In WSP, the predominant monosaccharides were identified as arabinose and galactose, followed by rhamnose, xylose, glucose (Figure 4A). CSP and ASP pectin fractions of longan aril tissues were rich in more than 70% of arabinose, followed by rhamnose and galactose (Figure 4B,C). Rhamnogalacturonan-I (RG-I) is the major component of the primary cell wall and middle lamella of dicotyledonous plants, which consists of the backbone of the repeating disaccharide units á-GalA-(1→2)-á-Rha RGs are usually branched with several neutral polymers such as arabinans, galactans, and arabinogalactans [4]. According to the content of neutral sugars in TFA-hydrolysates, the major component of pectin polysaccharides in longan aril tissues is hairy rhamnogalacturonan-I highly branched with arabinose and galactose side-chains.

**Figure 5.**
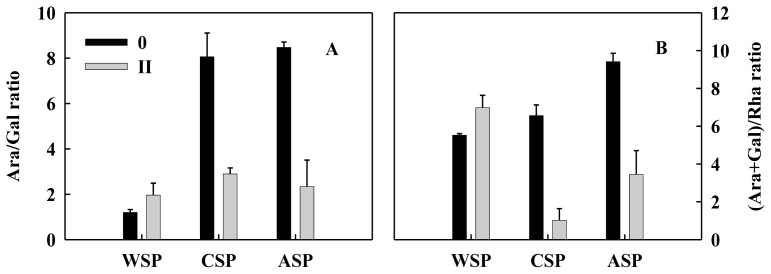
Ara/Gal (**A**) and (Ara + Gal)/Rha (**B**) ratios in different pectin polysaccharide fractions of 90 longan fruit with different breakdown indices (0, no aril breakdown; II moderate aril breakdown). Data were presented as means ± standard errors (*n* = 3).

The changes in monosaccharide compositions can reflect the modification characteristics of cell wall polysaccharide [11]. In pectin polysaccharides, Rha residues are branched points for the attachment of neutral sugar side chains, while Ara or/and Gal are the main constituents of branched arabinans, branched arabinogalactans, and linear galactanss of the rhamnogalacturonan-I [4]. In the present study, the molar percentages of Ara decreased largely in CSP and ASP from no aril breakdown to moderate aril breakdown while those of Rha apparently increased (Figure 4B,C). By contrast, the molar percentage of Ara increased, but that of Rha decreased in WSP (Figure 4A). Furthermore, Ara/Gal and (Ara + Gal)/Rha ratios in CSP and ASP pectin fractions decreased, but increased in WSP, from no aril breakdown to moderate aril breakdown (Figure 5A). The removal of neutral sugars, mainly Gal and/or Ara, from the side-chains is a mechanism of depolymerization of pectin polysaccharides in fruit [26]. Ara/Gal and (Ara + Gal)/Rha ratios can reflect the relative importance of Ara or Gal in side-chains of pectin, and that of neutral side-chains to the RG backbone in the depolymerization of pectin, respectively [14,27]. These results suggest that the degradation of WSP pectin fraction mainly occurred in the backbone of RG-I whereas the depolymerization of Ara-rich side-chains played a more important role in degradation of CSP and ASP.

It is well known that pectolytic enzymes such as PG, PME and PL are responsible for the degradation of pectin and pectic substances, which act on the main polyuronide chains of pectins and eventually cause cell lysis [4]. However, molecular evidence showed that expression of a chimeric polygalacturonase gene in transgenic rin (ripening inhibitor) tomato fruit results in polyuronide degradation but not fruit softening [28]. The other enzymes such as β-galactanase, β-galactosidase and α-arabinosidase act on the side chains of the galacturonide backbone, eventually degrading the entire pectic substance [29–31]. Lin *et al.* found that protopectin content and activities of PG and pectinesterase (PE) decreased with increased aril breakdown in longan, whereas β-galactosidase activity increased [30]. The present study showed the depolymerization of CSP and ASP pectin polysaccharides happened predominantly in Ara-based side chains. These results implied that the degradation of side chains of pectin polysaccharides played a role in aril breakdown of longan fruit. For CSP and ASP pectin polysaccharides of longan aril, the main neutral sugar was Ara and a substantial amount of Ara was lost during aril breakdown, suggesting the involvement of arabinanase, which possibly was associated with aril breakdown of longan fruit. However, further investigation on the change in arabinanase activity is needed to confirm the speculation.

As shown in Figure 4D, hemicellulose fraction from longan aril tissues contained high levels of xylose and glucose, followed by mannose and galactose. In addition, small amount of arabinose and rhamnose were found in hemicellulose. According to the molar compositions of monosaccharides, it is suggested that the hemicellulose of longan aril tissues consisted mainly of xyloglucan. Glucomannan and galactoglucomannan also might exist in hemicellulose of longan aril tissues. Similarly, high levels of xylose and glucose were observed in the monosaccharide composition of hemicellulose fractions in Japanese plum [14]. In the present study, the molar percentage of xylose of hemicelluose fraction increased largely while that of glucose decreased from no aril breakdown to moderate aril breakdown, suggesting that more backbones of xyloglucan were depolymerized. The modification and subsequent degradation of hemicelluloses requires an entire set of enzymes including endo-β-1,4-glucanase (EGase), xyloglucan endotransglucosylase-hydrolase (XTH), β-mannanases, β-mannosidases, β-xylanases and β-xylosidases [32–35]. Xiao *et al.* (2009) reported that EGase activity in aril tissues of longan fruit increased with the appearance of aril breakdown symptoms [36], which was consistence with the present result that the molar percentage of glucose significantly decreased during aril breakdown. It is suggested that modification of backbone of xyloglucan caused by EGase played a role in aril breakdown of longan fruit.

### 2.4. Molecular Mass Profile of Pectin and Hemicellulose Polysaccharide Fractions

**Figure 6.**
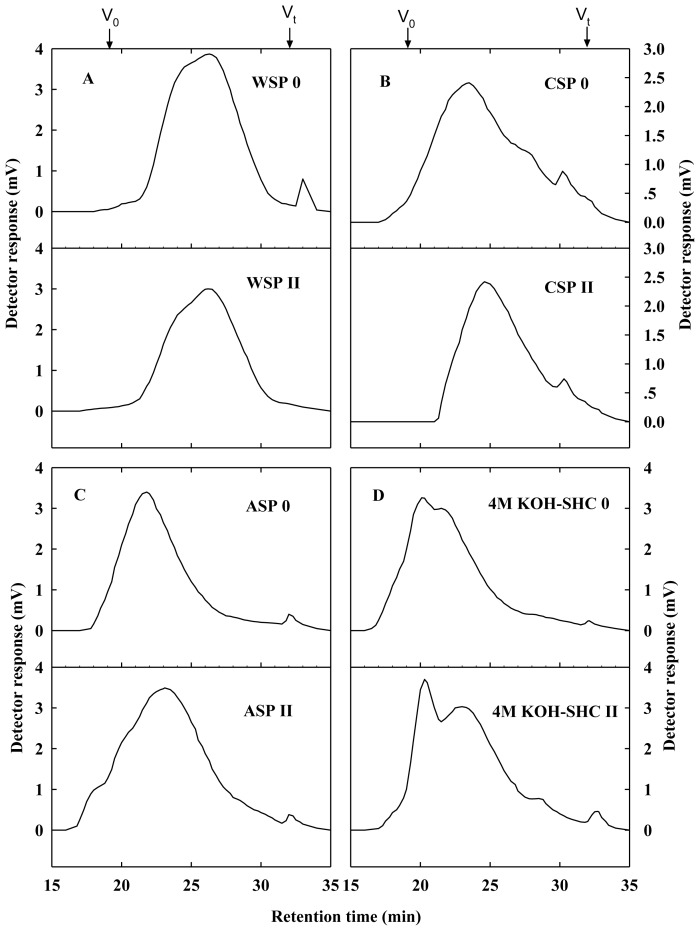
Gel permeation chromatography profiles of WSP (**A**), CSP (**B**), ASP (**C**) and 4 M KOH-SHC (**D**) polysaccharide fractions from aril tissues of longan fruit with different breakdown indices (0, no aril breakdown; II moderate aril breakdown). *V*_0_, void volume; *V*_t_, total volume.

The molecular mass distributions of pectin and hemicellulose polysaccharide fractions from longan aril tissues were analyzed by gel permeation chromatography (GPC). In this experiment, the retention times (RT) of the standard dextrans of mean molecular weights of 5.2, 11.6, 23.8, 48.6, 148, 273, 410, 668, 1400 kD were 30.82, 29.35, 27.28, 25.62, 23.52, 22.20, 21.76, 21.31 and 20.64 min, respectively. As shown in Figure 6, three pectin fractions of longan aril tissues showed differential changes in molecular weight distribution during texture deterioration. No obvious molecular weight decrease was observed in WSP pectin fraction from no aril breakdown to moderate aril breakdown. However, the molecular weight distributions of CSP and ASP pectin fractions showed a large decrease during texture deterioration. These results confirmed the depolymerization of pectin polysaccharide in longan fruit during texture deterioration and the depolymerization mainly occurred in ionically bound CSP and covalently bound ASP.

For 4 M KOH-SHC polysaccharide fraction, one eluted peak with lower molecular weight appeared in longan fruit with moderate aril breakdown. Depolymerization of matrix glycans is at least as important as that of pectin polysaccharide, which leads to the loss of structural integrity of cell wall [14,19,22]. Some studies have showed the disassembly of hemicelluloses in ripening fruits such as banana [9], peach [23] and plum [14]. In banana, the depolymerization took place only in tightly bound glycans [9]. Our results also demonstrated that tightly bound matrix glycans underwent apparent depolymerization in longan fruit during storage, which possibly was related to the aril breakdown.

## 3. Experimental Section

### 3.1. Plant Materials

Longan (*Dimocarpus longan* Lour. cv. Shixia) fruit were harvested at commercial mature stage from an orchard in Guangzhou in 2012. The maturity was determined according to total soluble solids (TSS) content. Fruit were selected for uniformity of shape, color and size, and any blemished or diseased fruit were discarded. A total of 1350 fruit were used. All fruit were dipped in 0.1% TBZ (thiabendazole) for 3 min and air-dired for 1 h at 25 °C, then packed in 0.03 mm thick polyethylene bags (90 fruit per bag) and finally stored in a constant temperature (25 °C) and humidity (90% ± 5% RH.) incubator. Sub-samples were taken for evaluation and classification of aril breakdown Replication with 90 fruit was three-fold (*n* = 3) for each time.

### 3.2. Evaluation of Aril Breakdown

**Figure 7.**
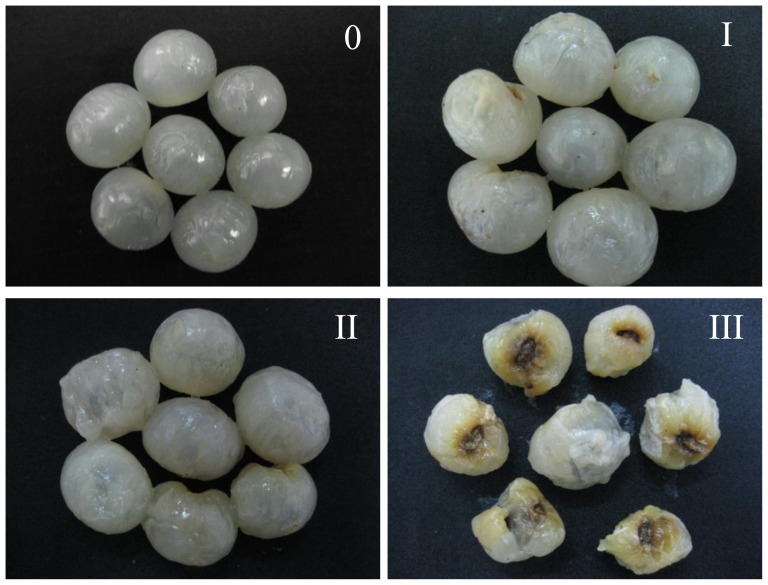
Visual appearance of different aril breakdown indices of longan fruit.

There is no an objective way of assessing aril breakdown. In this study, a subjective way was used to assess aril breakdown by a panel of testers. The panel comprised of nine members, aged 30–50 years, and with quality evaluation experience in fruits and vegetabes, evaluated aril breakdown according to the following scale: 0, no aril breakdown; I, slight aril breakdown; II, moderate aril breakdown; III, severe aril breakdown, as shown in Figure 7. The aril breakdown index was calculated using the following formula: ∑ (aril breakdown scale × proportion of corresponding fruit within each scale). Although the results from different researchers possibly exist slight differences, the method is in wide use.

### 3.3. Preparation of Cell Wall Material (CWM)

CWM was extracted according to the method of Fry [37]. The frozen pulp tissues (100 g) were blended in 300 mL of 95% (*v*/*v*) ethanol using a homogenizer. Endogenous enzymes were inactivated by incubating in boiling water for 10 min. After centrifugation, the residue was washed sequentially with 200 mL of mixture solution of chloroform: methanol (1:1, *v*/*v*) and 200 mL of acetone. After that, the starch was removed by re-extracting overnight in 90% DMSO. Following centrifugation, the precipitate was washed twice with 70% ethanol, then filtered and finally dried in the vacuum oven at 40 °C. The dried powder was considered as cell wall material (CWM). Each aril breakdown scale comprised three duplicates and 60 fruit were used for each duplicate.

### 3.4. Fractionation of Pectin and Hemicellulose Polysaccharides

Different fractionations of cell wall polysaccharides from longan aril CWM were extracted according to the method of Selvendran and Ryden [38] with some modification. About 5 g of CWM was stirred with 200 mL of distilled water overnight at room temperature. After centrifugation, the supernatant was lyophilized and designated as water-soluble pectin (WSP). The residue was then extracted sequentially with 50 mM CDTA in 50 mM sodium acetate (pH 6.0), 50 mM Na_2_CO_3_, and 4 M KOH to produce CDTA-soluble pectin (CSP), alkali soluble pectin (ASP), 4 M KOH-soluble hemicellulose (4 M KOH-SHC), respectively. For each extraction, the residues were stirred for 24 h in the presence of 0.02% sodium azide and then centrifuged for 15 min at 4000 × *g*. The supernatants were dialyzed (*M*_w_ cutoff 3500) exhaustively against distilled water at 4 °C and then lyophilized. For 4 M KOH-SHC determination, the supernatants were neutralized with glacial acetic acid prior to dialysis and lyophilization.

### 3.5. Analysis of Monosaccharide Compositions

The monosaccharide compositions of pectin and hemicellulose polysaccharides were analyzed by the method of Wen *et al.* [39]. Briefly, 10 mg of sample was hydrolyzed with 10 mL of 2 M trifluoroacetic acid (TFA) at 120 °C for 6 h. The mixture was dried and then incubated with 2 mL of pyridine, 0.4 mL of hexamethyldisilazane and 0.2 mL of trimethychlorosilane for 30 min. After trimethylsilylation, the derivatives were loaded onto a GC-2010 gas chromatograph (Shimadzu Corporation, Shanghai, China) equipped with a TRX-5 capillary column and a flame ionization detector (FID), using inositol as the internal standard. The operation conditions were followed as: injection temperature: 250 °C; detector temperature: 290 °C; column temperatures programmed from 130 to 180 °C at 2 °C/min, holding for 3 min, then increased 220 °C at 10 °C/min, holding for 3 min and finally increased to 280 °C, holding for 10 min. The sugars (arabinose, rhamnose, xylose, galactose, glucose, mannose, fucose) were used as standards. The molar percentage of different monosaccharide in total amount of seven monosaccharides was calculated using the external standards.

### 3.6. Gel-Permeation Chromatography

Molecular weight distributions of pectin and hemicellulose polysaccharides fractions from longan aril tissues were analyzed by a high-performance gel permeation chromatography (GPC) on a HPLC (Waters 1525, Waters Corp., Milford, MA, USA) equipped with TSK-GEL Guard Column (PWXL 6.0 × 0 mm), TSK-GEL 4000K gel column (PWXL 7.8 × 300 mm) (TOSOH Corp., Tokyo, Japan), and Waters 2414 Refractive Index Detector. The detailed operation conditions were as follows: mobile phase: 0.2 mol/L phosphate buffer (pH 7.0); flow rate: 0.6 mL/min; column temperature: 35 °C; injection volume: 30 μL; running time: 45 min. The dextrans with various molecular weights (5.2, 11.6, 23.8, 48.6, 148, 273, 410, 668, 1400 kD, Pharmacia, Stockholm, Sweden) were used as standards to calibrate the column and fit the regression curve. Breeze GPC software was employed to calculate the molecular weight. The molecular weight was determined according to the equation of elution volume and the logarithm of their molecular weights.

### 3.7. Data Handling

Experiments were designed with three replicates. Data were tested by analysis of variance using SPSS (Version 13.0, SPSS Inc., Chicago, IL, USA, 2005). Least significant differences (LSDs) were calculated to compare significant effects at the 5% level.

## 4. Conclusions

In conclusion, the remarkable changes in the composition and molecular mass of three pectin and one hemicellulose fractions occurred in harvested longan fruit during storage. During aril breakdown, CSP content increased, accompanied by the decrease of ASP and 4 M KOH-SHC polysaccharides, which might be related to the solubilization and conversion of cellular wall components. Moreover, the monosaccharide composition profiles of pectin and hemicellulose polysaccharides showed that the depolymerization of CSP and ASP pectin polysaccharides occurred predominantly in side-chains formed of Ara residues while more backbones were depolymerized in WSP and 4 M KOH-SHC polysaccharide. Gel permeation chromatography analysis further revealed that the molecular weight distribution of CSP, ASP and 4 M KOH-SHC polysaccharides tended to downshift. Overall, both enhanced depolymerization and structural modifications of polysaccharides in the CSP, ASP and 4 M KOH-SHC fractions might be responsible for aril breakdown of harvested longan fruit.
